# Palmitic acid declines glucose uptake in HepG2 cells via modulating phosphoglucomutase 1 to repress phosphatidylinositol 3 kinase/protein kinase B and JNK pathways via inducing microRNA-124-3p

**DOI:** 10.55730/1300-0152.2618

**Published:** 2022-04-25

**Authors:** LingHui ZHANG, ShengLi ZHANG

**Affiliations:** 1 Department of Endocrinology, Hubei Third People’s Hospital affiliated to Jianghan University, Wuhan City, China; 2 Department of Cardiovascular Medicine, Hubei Third People’s Hospital affiliated to Jianghan University, Wuhan City, China

**Keywords:** Palmitic acid, microRNA-124-3p, phosphoglucomutase 1, PI3K/AKT, JNK, insulin resistance, autophagy, cell proliferation, inflammatory response

## Abstract

Diabetes resulting from insufficient insulin secretion or insulin resistance (IR) is a highly prevalent metabolic disease. Since microRNAs have been linked with elevated IR, the current research hypothesized that miR-124-3p has a role in IR and the establishment of IR and type 2 diabetes (T2DM). The study aimed to explore the molecular mechanisms of miR-124-3p which influence IR leading to T2DM establishment. HepG2 cells were cultured in vitro, and palmitic acid (PA) was used to construct the IR cell model. In the IR model, transfection of miR-124-3p or phosphoglucomutase 1 (PGM1) linked plasmids were transfected into HepG2 cells. RT-qPCR was used to determine the miR-124-3p and PGM1 expressions in the cells. Cell viability was assessed through CCK-8 assays, while glucose consumption was studied using a glucose uptake test. Interaction between miR-124-3p and PGM1 was examined using a dual-luciferase reporter assay. Autophagy, phosphatidylinositol 3 kinases (PI3K)/protein kinase B (AKT) and JNK pathways-linked factors, glucose transporter 4 (GLUT4), and c-Jun were determined through western blotting assays. MiR-124-3p expression was elevated, but PGM1 was reduced in the IR model. Glucose uptake was reduced posttreatment with 0.8 mM PA. There was a significantly increased PI3K, p-PI3K, AKT, p-AKT, GLUT4, LC3I/II, Beclin-1, p-JNK1/2, and c-Jun, but reduced p62 expressions were presented in the PA + miR-124-3p inhibitor compared to the PA and PA + inhibitor NC groups. PGM1 binds directly to miR-124-3p through the 3′ UTR region target. Overall, miR-124-3p downregulates glucose consumption via targeting PGM1 to repress PI3K/AKT and JNK pathways. Silencing PGM1 inhibited the suppressor role of miR-124-3p on glucose uptake, cell proliferation, and inflammation. In conclusion, miR-124-3p reduces glucose uptake in HepG2 cells via PGM1/PI3K/AKT modulation. MiR-124-3p targets PGM1 in IR and may provide an effective therapeutic alternative for T2DM.

## 1. Introduction

Diabetes resulting from insufficient insulin secretion or insulin resistance (IR) is a highly prevalent metabolic disease worldwide (Bartel et al., 2004). According to the latest data, over 463 million adults are diagnosed with diabetes, which is expected to rise to 700 million by 2045 (Zheng et al., 2018). Diabetes is categorized into type 1 diabetes (T1DM), type 2 diabetes (T2DM), and gestational diabetes (GDM). T2DM is the most common, with over 90% of all diabetes cases (Galicia-Garcia et al., 2020). Impaired insulin signaling pathway, systemic IR, and absence of response to insulin target cells (like liver cells, fat cells, and skeletal muscle cells) are the major factors leading to T2DM (Saltiel et al., 2001). IR is present during the progression of T2DM, and the metabolism in T2DM patients can be mitigated through IR reduction (Tahrani et al., 2011). The pathogenesis of IR is extremely complex, and according to a previous study, increased free fatty acids (FFA) can result in IR in the muscle and liver tissues (Samuel et al., 2010). However, the molecular mechanisms linking FFA and IR remain unclear. Palmitic acid (PA) is a classic saturated free fatty acid found in animals and plants (Gao et al., 2010). PA has been successfully induced in IR models in cultured HepG2 cells (Ishii et al., 2015), (Charytoniuk et al., 2019).

According to previous reports, various microRNAs (miRNAs) have roles in the cascade of main proteins in the insulin pathway that impact IR, for instance, let-7f and insulin growth factor-1 receptor (Hu et al., 2014), let-7a, and phosphatidylinositol 3 kinase (PI3K)/protein kinase B (AKT) (Wang et al., 2019), miR-133a and glucose transporter 4 (GLUT4) (Horie et al., 2009). Activated insulin receptor substrate 2 induces the PI3K/AKT pathway to transduce insulin activity, subsequently activating the absorption of glucose via the type 4 insulin-sensitive GLUT4 in the plasma membrane (Evans et al., 2005). MiR-124-3p negatively regulates the differentiation of hepatocytes into insulin-producing cells (IPCs) in vitro (Pan et al., 2020). However, the role of miR-124-3p in T2DM is still unknown. Phosphoglucomutase 1 (PGM1) is crucial in modulating insulin secretion (Galcheva et al., 2018) and implicated in congenital hyperinsulinemia (Gϋemes et al., 2020), but the mechanism and role of PGM1 in T2DM have not been investigated.

The study hypothesized that miR-124-3p plays a role in the IR and establishment of T2DM via the miR-124-3p/PGM1 axis. The study aimed to determine the expression of miR-124-3p in the insulin-resistant HepG2 cells model, assess whether PGM1 is a target gene of miR-124-3p, and understand the effects of miR-124-3p in proliferation and autophagy of HepG2 cells. Finally, the effect of miR-124-3p inhibition on PGM1 and T2DM progression was investigated. The results confirmed that miR-124-3p inhibits glucose uptake in HepG2 cells, miR-124-3p facilitates cell autophagy but represses inflammation via depressing the PI3K/AKT and JNK pathways, miR-124-3p targets PGM1 and miR-124-3p reduces glucose uptake via targeting PGM1 to repress PI3K/AKT and JNK pathways.

## 2. Materials and methods

### 2.1. Cell culture

The HepG2 cells used in the study were purchased from American Type Culture Collection (ATCC) (Manassas, VA, USA). The cells were maintained in Dulbecco’s Modified Eagle Medium (DMEM) (Gibco, NY, USA) supplemented with 10% fetal bovine serum, 100 U/mL penicillin, and 0.1 mg/mL streptomycin at 37 °C and 5% CO_2_. The cells were passaged in the logarithmic growth phase at 70%–80% confluence.

### 2.2. Glucose uptake test

The HepG2 cells were transferred to a 6-well plate and treated for 24 h with 0–0.8 mM PA. The glucose uptake was assessed through the glucose oxidase assay (Bio-Rongsheng, Shanghai, China) following the manufacturer’s instructions. The resulting solution’s absorbance at a wavelength of 490 nm was proportional to glucose concentration.

### 2.3. Cell viability determination

The Cell Counting Kit-8 (CCK-8) assay kit (Dojindo Molecular Technologies, Inc.) was used to detect cell viability. The HepG2 cells were seeded into a 96-well plate at a density of 6000 cells per well, and treatment was as described above. Cells were then incubated with 10% CCK-8 solution, and the absorbance was measured at 450 nm in a microplate reader (Thermo Fisher Scientific, Inc., USA). Each experiment was performed independently in triplicate.

### 2.4. Cell transfection

The inhibitor negative control (NC), miR-124-3p inhibitor (100 nM)(Sangon Biotech, Shangai, China), and si-NC/PGM1 (GenePharma Co. Ltd., Shanghai, China) were transfected into HepG2 cells using the lipofectamine 2000 transfection kit (Thermo Fisher Scientific, Waltham, MA, USA) as per the instructions of the manufacturer. The successfully transfected cells were cultured in serum-free DMEM and cultivated further.

### 2.5. EdU assay

The BeyoClick™ EdU-555 cell proliferation detection kit (Shanghai Biyuntian Biotechnology Co., Ltd., Shanghai, China) was used to study cell proliferation. The 2X EdU working solution was preheated at 37 °C and added to the well plate in equal volume with the original culture solution to obtain a final EdU concentration of 10 μM and finally incubated. The culture medium was discarded and 1 mL of 4% paraformaldehyde fixative was added and incubated. The fixative was then removed and permeabilization solution (1 mL) made of 0.3% Triton X-100 in phosphate buffer saline (PBS) was added to each well and further incubated. The solution was discarded, and Click additive was dissolved in deionized water and stored at −20 °C. For the preparation of the Click reaction solution, each well required: Click reaction buffer (430 μL), CuSO4 (20 μL), Azide (5551 μL), and Click additive solution (50 μL). The Click reaction solution (0.5 mL) was added to each well to ensure the reaction solution evenly covered the sample. The sample was incubated, and later, the Click reaction solution was aspirated. The 4′, 6-diamidino-2-phenylindole (DAPI) (1000X) was diluted with PBS at a ratio of 1: 1000, and 1 mL was added to each well for nuclear staining. The 1X DAPI solution was then discarded, and the sample was examined under a fluorescence microscope.

### 2.6. Quantitative real-time polymerase chain reaction (qRT-PCR)

The HepG2 cells were harvested after the appropriate treatment and washed thrice in ice-cold phosphate-buffered saline. TRIzol reagent (Life Technologies, NY, USA) was used to extract total RNA from HepG2 cells, and the concentration and purity of RNA were determined using an ultra-micro spectrophotometer NanoDrop2000 (Thermo Fisher Scientific, USA). Reverse transcription of total RNA was performed using Revert Aid First Strand cDNA Synthesis Kit (Thermo Fisher Scientific, USA) using 1 μg of the total RNA and Oligo (dT) primers.

The cDNA was then synthesized through reverse transcription using TaKaRa Reverse Transcription Kit (Takara, Tokyo, Japan). The cDNA (1 μL) template was prepared using 0.4 μL of RT-FP (10 μmol/L), 0.4 μL of RT-RP (10μmol/L), 8.2 μL of RNA-free enzyme water, and 10 μL of 2X SYBR Green Mix in a fluorescent quantitative PCR analyzer (CFX Connect, USA). The PCR conditions were done at 95°C for 30 s, 40 cycles of denaturation at 95°C for 5 s, then annealing at 60 °C for 30 s, extension at 95 °C for 5 s, and a final extension at 60 °C for 60 s. β-actin was employed as an internal control to standardize RNA expression, while U6 was an internal reference for miRNA. The relative fold changes of the genes were analyzed by the 2^-ΔΔCt^ method. The amplification primer sequence of each gene and its primer was detailed in Table.

### 2.7. The luciferase activity assay

miRanda (www.microrna.org), PicTar (pictar.mdcberlin.de/), and TargetScan (www.targetscan.org) were used for the prediction of miR-124-3p target genes. The PGM1 *3*′-untranslated region (*3*′-*UTR*) was amplified to gain mutant sequences using a site-directed mutation kit (NBS company, Beijing, China). The amplified PGM1 3′-UTR wild-type (WT) and mutant (MT) sequences were inserted into the psi-CHECK2 fluorescent reporter gene plasmid (Promega Company, USA). A luciferase reporter assay was used to validate the prediction. Later, WT and MT PGM1 3′-UTR plasmid, miR-124-3p mimic (100 nM), miR-124-3p inhibitor ((100 nM), and its negative control were separately transfected into the cells using LipofectamineTM 3000 (Thermo Fisher Scientific, USA) transfection reagent following the manufacturer’s instructions. The cells were harvested 48 h later, and the luciferase activity was assessed through Dual-Luciferase Reporter Assay System (Promega, Shanghai, China). All the assays were done in triplicates.

### 2.8. Western blot analysis

After a successful transfection, the HepG2 cells were lysed in RIPA lysis buffer and PMSF at low temperatures. The total protein concentration was detected using a BCA assay kit (Novizan, Nanjing, China). The protein (30 μg/lane) was separated using 10% sodium dodecyl sulfate-polyacrylamide gel electrophoresis (SDS-PAGE), and the electroblot was transferred onto a polyvinylidene fluoride membrane (Millipore, Billerica, MA, USA). The membrane was then blocked for 1 h with 5% skim milk diluted in tris-buffered saline of 0.1% Tween-20. The membrane was then incubated in with the rabbit antihuman PGM1 (ab192876, PI3K (ab191606), p-PI3K (ab278545), AKT (ab18785), p-AKT (ab81283), GLUT4 (ab33780), Beclin-1 (ab210498), LC3I/II (ab192890), p62 (ab109012), JNK1/2 (ab4821), p-JNK1/2, c-Jun (ab31419) primary antibodies, all from Abcam (Cambridge, UK) (all 1: 1000) and horseradish peroxidase-labeled goat antirabbit immunoglobulin G (IgG) (1: 5000, Solarbio Technology Co., Ltd.). The chemiluminescence imaging system (Shanghai Tianneng Technology Co., Ltd., Shanghai, China) was used to visualize and analyze the bands. β-actin (4970S, 1: 1000, Cell Signaling Technology, Boston, USA) was used as a sample loading control, and the experiment was performed three times independently.

### 2.9. Statistical analysis

Statistical analysis was performed with GraphPad Prism 8 software, and all the data were presented as mean ± standard deviation. The t**-**test was used for two-group comparison, while a one-way analysis of variance was used to compare multiple groups. Dunnett’s multiple comparisons test was used for post hoc multiple comparisons. p < 0.05 was considered statistically significant.

## 3. Results

### 3.1. MiR-124-3p inhibits glucose uptake in HepG2 cells

**Figure 1:**
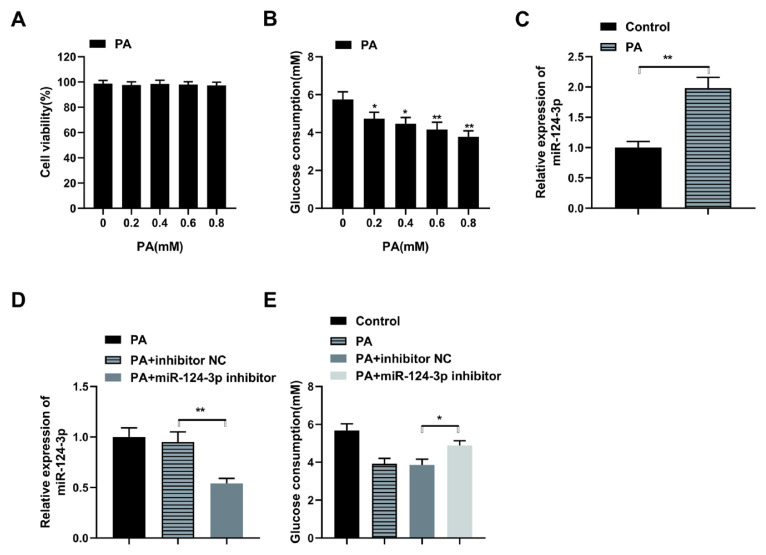
MiR-124-3p inhibits glucose uptake in HepG2 cells. (A) Assessment of cell viability in IR HepG2 cell model through CCK-8, after treatment with various concentrations of palmitic acid (PA); (B) The glucose uptake test done through glucose oxidase method. Various concentrations of PA were used in detecting the glucose uptake capacity; (C) qRT-PCR to detect miR-124-3p mRNA expression following the induction of PA (0.8 mM); (D) qRT-PCR to detect miR-124-3p mRNA expression in PA, PA+ inhibitor NC, or PA+miR-124-3p inhibitor transfected cells. (E) Determination of Glucose consumption in PA, PA+ inhibitor NC or PA+miR-124-3p inhibitor transfected cells. * p < 0.05, ** p < 0.01.

The IR model was treated with PA to determine the effects of miR-124-3p on T2DM. According to the results, the viability of the cells was not affected following the treatment with 0.2 to 0.8 mM PA (Figure 1A). However, the glucose uptake was significantly reduced following the treatment with 0.8 mM PA (Figure 1B, p < 0.05). Consequently, a concentration of 0.8 mM PA was chosen to induce IR in HepG2 cells. Next, the mRNA expression of miR-124-3p was determined in the PA-treated and control cells through RT-PCR. The observations confirmed a significantly elevated miR-124-3p mRNA expression in the PA-treated compared to the control cells (Figure 1C, p < 0.01). Furthermore, miR-124-3p mRNA expression was significantly reduced in the PA+miR-124-3p inhibitor compared to the PA+ inhibitor NC or the PA group (Figure 1D, P < 0.01), indicating a successfully silenced miR-124-3p. Moreover, the glucose uptake assay confirmed a significantly increased glucose consumption in the PA+miR-124-3p inhibitor compared to the PA+ inhibitor NC or the PA groups (Figure 1E, P < 0.05), indicating that miR-124-3p suppressed the glucose uptake in HepG2 cells. These observations confirmed that miR-124-3p is elevated in the HepG2 IR model, consequently reducing the glucose uptake in HepG2 cells.

### 3.2. MiR-124-3p facilitates cell autophagy, but repressed inflammation via depressing the PI3K/AKT and JNK pathways

**Figure 2:**
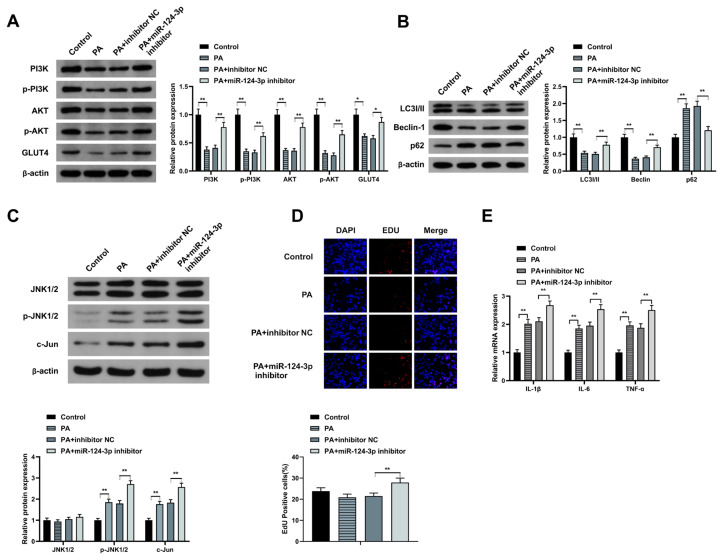
MiR-124-3p facilitates cell autophagy but repressed inflammation via depressing the PI3K/AKT and JNK pathways. Western blot assays to detect; (A) PI3K/p-PI3K, AKT/p-AKT, GLUT4; (B) autophagy-related markers of LC3I/II, Beclin-1, p62, and (C) JNK1/2, p-JNK1/2, and c-Jun; (D) EDU assay for the assessment of cell proliferation in PA, PA+ inhibitor NC or PA+miR-124-3p inhibitor transfected cells; (E) qRT-PCR assessment of inflammatory cytokines IL-1β, IL-6, and TNF-α. A-E, in HepG2 cells of each group. * p < 0.05; ** p < 0.01.

The possible role of miR-124-3p in the PI3K/AKT pathway was investigated through western blotting. The results confirmed a significantly increased PI3K/AKT pathway-linked proteins in the PA+ miR-124-3p inhibitor compared to the PA and PA + inhibitor NC groups (Figure 2A), confirming that miR-124-3p repressed PI3K/AKT pathway. The role of miR-124-3p on autophagy was investigated through western blotting. The results showed that miR-124-3p enhanced autophagy (Figure 2B). Contrastingly, p-JNK and c-Jun expressions were significantly elevated (Figure 2C), indicating that miR-124-3p repressed PI3K/AKT pathway. The proliferative capacity of HepG2 cells was investigated following the transfection with PA+miR-124-3p inhibitor, PA+ inhibitor NC, PA, or the control plasmids. The observations confirmed significantly increased proliferation of the PA+miR-124-3p inhibitor group compared to the NC or the controls (Fig 2D). Furthermore, the role of the expression of inflammatory cytokines was investigated in the HepG2 cells transfected with PA+miR-124-3p inhibitor, PA+ inhibitor NC, PA, or the control plasmids. According to the observations, IL-1β, IL-6, and TNF-α cytokines were significantly elevated in the PA+miR-124-3p inhibitor group than in the negative controls (Figure 2E). Taken together, these observations indicate that miR-124-3p facilitated cell autophagy but repressed proliferation and inflammation via the PI3K/AKT and JNK pathways.

### 3.3. MiR-124-3p targets PGM1

**Figure 3:**
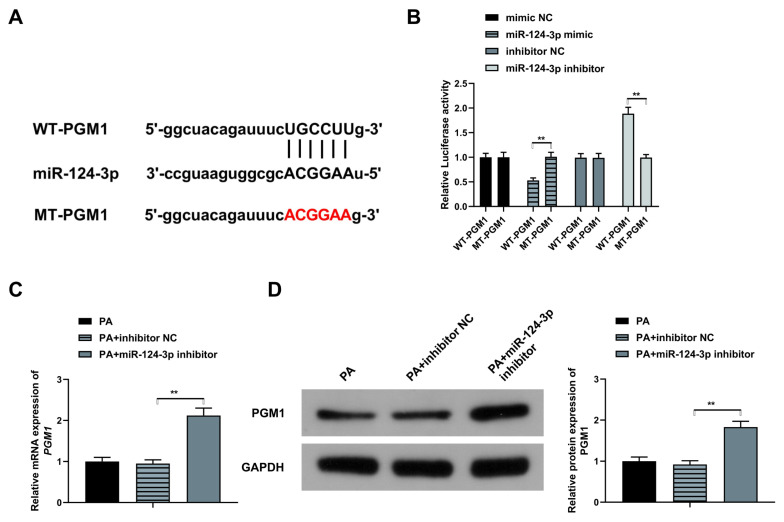
MiR-124-3p targets PGM1. (A) miRanda (www.microrna.org), PicTar (pictar.mdc-berlin.de/), and TargetScan (www.targetscan.org) were used to predict the design of the target site and mutation site of miR-124-3p and PGM; (B) Luciferase activity assay and determination of the relative activity of each plasmid luciferase; (C, D) qRT-PCR and Western blot to detect PGM1 gene expression in each group of HepG2 cells. ** p < 0.01.

PGM1 has a role in congenital hyperinsulinemia. We thus speculated that miR-124-3p negatively modulates PGM1, inhibiting glucose uptake in HepG2 cells. To explore the mechanism of miR-124-3p, miRanda (www.microrna.org), PicTar (pictar.mdc-berlin.de/), and TargetScan (www.targetscan.org) were used for the prediction of miR-124-3p target genes. According to the findings, miR-124-3p directly binds with PGM1 in the 3′UTR region, as demonstrated in Figure 3A. Furthermore, the luciferase activity was significantly increased in the MT-PGM1+ miR-124-5p mimic compared to the WT-PGM1 miR-124-5p mimic but significantly reduced in the MT-PGM1+miR-124-5p inhibitor compared to the WT-PGM1 +miR-124-5p inhibitor transfected cells (p < 0.01) (Figure 3B). The use of RT-qPCR and western blot to determine the PGM1 mRNA and protein expression confirmed significantly elevated PGM1 levels in PA+miR-124-3p inhibitor compared to PA+ inhibitor NC (Figures 3C and 3D, P < 0.01). In summary, these observations showed that PGM1 is a target of miR-124-3pin T2DM.

### 3.4. MiR-124-3p reduces glucose uptake via targeting PGM1 to repress PI3K/AKT and JNK pathways

**Figure 4:**
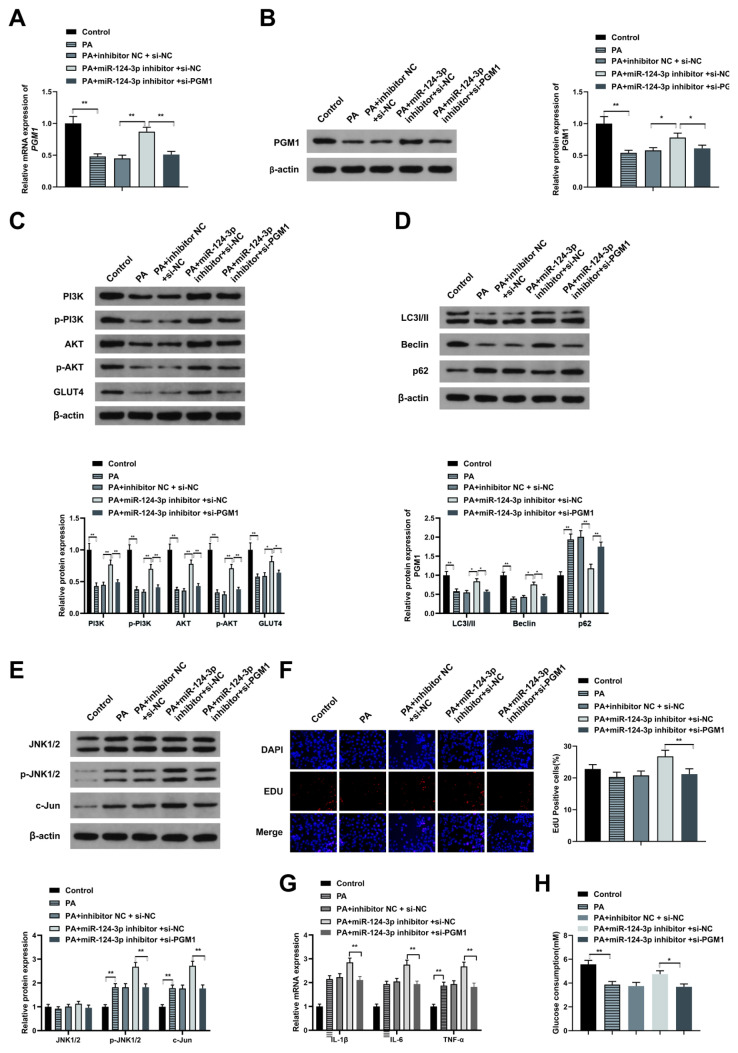
MiR-124-3p reduces glucose uptake via targeting PGM1 to repress PI3K/AKT and JNK pathways. (A) qRT-PCR for the determination of mRNA PGM1 expression in PA+miR-124-3p inhibitor + si-PGM1, PA+miR-124-3p inhibitor + si-NC, PA+ inhibitor NC + si-NC, PA or control cells; (B) Western blot to detect PGM1 gene expression in PA+miR-124-3p inhibitor + si-PGM1, PA+miR-124-3p inhibitor + si-NC, PA+ inhibitor NC + si-NC, PA or control cells; (C) Western blot to detect PI3K/p-PI3K, AKT/p-AKT, GLUT4 gene expression in PA+miR-124-3p inhibitor + si-PGM1, PA+miR-124-3p inhibitor + si-NC, PA+ inhibitor NC + si-NC, PA or control cells; and (D) LC3I/II, Beclin-1, p62, (E) JNK1/2, p-JNK1/2, and c-Jun genes expression; (F) EDU to detect the proliferation ability in in PA+miR-124-3p inhibitor + si-PGM1, PA+miR-124-3p inhibitor + si-NC, PA+ inhibitor NC + si-NC, PA or control cells; (G) qRT-PCR assessment of proinflammatory cytokines IL-1β, IL-6 and TNF-α in in PA+miR-124-3p inhibitor + si-PGM1, PA+miR-124-3p inhibitor + si-NC, PA+ inhibitor NC + si-NC, PA or control cells; (H) Glucose oxidase assay to detect the glucose uptake capacity. * p < 0.05; ** p < 0.01.

The mechanism of glucose consumption regulation by miR-124-3p was also investigated. It was hypothesized that miR-124-3p suppressed autophagy but enhanced glucose uptake in HepG2 cells via repressing PGM1. To confirm this theory, the HepG2 cells were separately cotransfected with PA + miR-124-3p inhibitor + si-NC, PA + miR-124-3p inhibitor + si-PGM1, PA + inhibitor NC + si-NC, PA or the control. The levels of PGM1 mRNA expressions were then assessed through RT-qPCR and western blotting. According to the results, the PGM1 expression was significantly reduced in the PA and the PA + miR-124-3p inhibitor + si-PGM1 compared to the controls and in the PA + miR-124-3p inhibitor + si-NC group (Figures 4A and 4B). Further observations confirmed that PGM1 reversed the suppression of miR-124-3p on the PI3K/AKT pathway (Figure 4C). Meanwhile, PGM1 also downregulated the autophagy effects of miR-124-3p (Figure 4D). Contrastingly, p-JNK and c-Jun were significantly elevated (Figure 4E, p < 0.01), indicating that PGM1 represses the inhibition of miR-124-3p on the PI3K/AKT pathway. Meanwhile, PGM1 restrained the suppression of miR-124-3p on cell proliferation, inflammation, and glucose uptake in HepG2 cells (Figures 4F–4H). No significant difference was observed between the PA and the PA inhibitor NC + si-NC. In summary, miR-124-3p reduced glucose consumption in HepG2 cells via targeting PGM1 to inhibit PI3K/AKT and JNK pathways.

## 4. Discussion

T2DM is a risk factor for various microvascular and macrovascular complications, leading to increased mortalities in T2DM patients (Lovis et al., 2008). Fasting blood glucose (FBG) and hemoglobin A1c (HbA1c) are important indicators used to screen and diagnose diabetes. However, only when the patient’s disease manifestations have resulted in metabolic changes can FBG and HbA1c indicators be used to screen and diagnose T2DM (Rezk et al., 2016). Most T2DM patients have increased plasma FFA levels proportional to the severity of insulin resistance. Food intake with increased saturated or mono-saturated fats is directly linked to high insulin resistance. High circulating FFAs levels initiate and promote the progression of insulin resistance. In this study, PA, a representative FFA, enhanced the expression of miR-124-3p in hepG2 cells. MiR-124-3p inhibited glucose uptake in HepG2 cells, miR-124-3p facilitated cell autophagy, but repressed inflammation via depressing the PI3K/AKT and JNK pathways, miR-124-3p targeted and bound PGM1 and miR-124-3p reduced glucose uptake via targeting PGM1 to repress PI3K/AKT and JNK pathways.

MiRNAs are small noncoding molecules found in human tissues and various body fluids such as peripheral blood (Tribolet et al., 2020). The miRNAs modulate gene expression and repress protein translation by combining with target genes’ 3′-untranslated region (UTR). As mediators of various aspects of oxidative stress, the focus on miRNAs has been increased (Bartel et al., 2004). The role and mechanisms of miR-124-3p in various diseases have been extensively studied. Overexpression of miR-124-3p has been shown to suppress MEKK3 expression and downregulate the p38MAPK signaling pathway expression, thus reducing the proliferation of macrophages and promoting apoptosis in coronary atherosclerotic mice model (Zhai et al., 2020). Li et al. also reported that osteocyte-derived exosomes containing miR-124-3p regulate Gal-3 expression in osteoblasts in high-glucose conditions, indicating a possible mechanism for diabetes mellitus-related pathologies of alveolar bone (Li et al., 2020). MiR-124-3p has been reported as a tumor suppressor against various tumors, including breast, pancreatic, gastric, brain, and prostate cancers (Jia et al., 2019).

In addition, miR-124-3p has a regulatory function in metabolism. For instance, it regulates liver fatty acid and cholesterol metabolism. In addition, miR-124-3p has been reported to have a close relationship with the differentiation of pancreatic cells and secretion of insulin through the targeting of Noc2 and Rab27A (Lovis et al., 2019). MiR-124-3p negatively modulates the differentiation of hepatocytes into IPCs in vitro. Downregulation of miR-124-3p has also been shown to influence the functioning of pancreatic-β-cell via the targeting of secreted frizzled-related protein 5 (SFRP5) in Diabetes mellitus (Jiang et al., 2021). In the current report, we demonstrate that microRNA-124-3p suppresses glucose uptake via phosphoglucomutase 1/phosphatidylinositol 3 kinase/protein kinase B and JNK axis in insulin resistant cell model.

The PI3K/AKT pathway is necessary for normal metabolism, and its imbalance results in obesity and T2DM (Huang et al., 2018). The mammalian kinase target of rapamycin (mTOR) is a negative regulator of autophagy. The PI3K/AKT/mTOR pathway activation enhances phosphorylation of mTOR downstream effector genes, further damaging autophagy activity (Liu et al., 2020). Previous studies have shown that the PI3K-Akt-mTOR pathway affects autophagy and mitigates IR (Song et al., 2021). Our findings also reported enhanced cell autophagy through miR-124-3p silencing via PI3K/AKT pathway inhibition. Proliferation was also downregulated via the suppression of PI3K/AKT and JNK pathways. In addition, Inflammation has been considered the major reason for IR (Shoelson et al., 2006), especially the activation of the JNK signal is implicated in the loss of insulin function (Solinas et al., 2017). Following the IR model construction, miR-124-3p was increased in the PA-treated cells, indicating that miR-124-3p might inhibit IR. Our findings, therefore, confirmed that miR-124-3p has a role in suppressing the proinflammatory cytokines IL-1β, TNF-α, and IL-6, thus protecting against IR.

In this research, PGM1 was confirmed as the immediate target of miR-124-3p. Through miR-124-3p silencing, PGM1 was negatively regulated by miR-124-3p in HepG2 cells. Further assays clarified that miR-124-3p exerts biological functions by targeting PGM1. PGM1 is implicated in glycogen metabolism and the reversible conversion of glucose 6-phosphate to glucose 1-phosphate (Tegtmeyer et al., 2014). AMP-activated protein kinase induces PGM1 by stimulating the phosphorylation of histone deacetylase 8 (HDAC8) (Li et al., 2020). Hence, it was hypothesized that HDAC8 is linked with miR-124-3p during IR.

The limitation of this study is that it only confirms in vitro data without any in vivo or clinical findings. The findings may not be a true reflection of what occurs in the clinical setting without further research. The use of one type of cell line is also a limitation of this study.

In conclusion, this work confirms the role of miR-124-3p in IR. Meanwhile, miR-124-3p facilitated autophagy but reduced inflammation, proliferation, and glucose uptake in HepG2 cells. Moreover, PGM1 was confirmed to be a target gene of miR-124-3p. MiR-124-3p facilitates autophagy but declines inflammation and glucose uptake in HepG2 cells via targeting PGM1/PI3K/AKT. The use of miR-124-3p to target PGM1 could be a novel and effective alternative for handling insulin resistance hence treating T2DM.

## Figures and Tables

**Table:** Primer sequence.

Name of primer	Sequences
MiR-124-3p-F	AAGTACTCTAAGGCACGCGGT
MiR-124-3p-R	CAGTGCAGGGTCCGAGGT
PGM1-F	AGCATTCCGTATTTCCAGCAA
PGM1-R	TCAGATTCCCAAAAAACTTCCAA
IL-1β-F	GGGGCGTCCTTCATATGTGT
IL-1β-R	ATACAACGGCTCCTCCGTTC
IL-6-F	GGGCTGCGATGGAGTCAGAG
IL-6-R	AGTGACTCAGCACTTTGGCAT
TNF-α-F	TGGGTAAGGAGATGCTTCCG
TNF-α-R	ATCGCGCGTTTGAAAGTGTC
U6-F	CTCGCTTCGGCAG CACA
U6-R	AACGCTTCACGAATTTGCGT
β-actin-F	CTCCATCCTGGCCTCGCTGT
β-actin-R	GCTGTCACCTTCACCGTTCC
